# Use of Vitamins K antagonists in non-valvular atrial fibrillation thromboembolic risk prevention in Burkina Faso

**DOI:** 10.11604/pamj.2016.24.108.7100

**Published:** 2016-05-31

**Authors:** Aristide Relwendé Yameogo, Jonas Koudougou Kologo, Germain Mandi, Hervé Poko Kabore, Georges Rosario Christian Millogo, Arthur André Taryetba Seghda, André Koudnoaga Samadoulougou, Patrice Zabsonre

**Affiliations:** 1 Department of Cardiology, University Hospital Yalgado Ouedraogo, Burkina Faso; 2 Training Unit and Research in Health Sciences (UFR / SDS), University of Ouagadougou, Burkina Faso

**Keywords:** Atrial fibrillation, stroke, vitamins K antagonists, Burkina Faso

## Abstract

**Introduction:**

Atrial fibrillation is the commonest cardiac rythm disorder. Thromboembolic accidents are common complications that should be prevented by anticoagulant treatment. The aim of our study is to assess the use of vitamins K antagonists in the prevention of thromboembolic risk in atrial fibrillation.

**Methods:**

It was a descriptive retrospective study of patients folders, performed in the cardiology department from January 1st 2010 to December 31st 2011. The study included all patients with non valvular atrial fibrillation. Thromboembolic risk was assessed through the CHA2DS2VASc score, and hemorrhagic risk through the HAS-BLED score.

**Results:**

Atrial fibrillation accounted for 10.6% of all hospitalizations (103/970). Five patients had contra indication to anticoagulants. Non valvular AF was noticed in 68 cases (66%). The non valvular AF was chronic in 40 cases (59%) and paroxystic in eight cases (12%). The median age of the population was 64.5+13.8 years old. Median CHA2DS2VASc score was 3.9 + 1.6. Two patients had a score < 1. Sex, place of residence, age > 65, and cardiac failure did not interfere with prescription of vitamins K antagonists. Ischemic stroke and intra cavity thrombus were the indications for vitamins K antagonists’ prescriptions. The median HAS-BLED score was 3.5 + 1.5. The rate of vitamins K antagonists use was 35.3%. One case of death due to hemorrhagic stroke was noticed.

**Conclusion:**

Guidelines on thromboembolic risk prevention are poorly used in the cardiology department. But the use of scoring systems allows the assessment of vitamins K antagonists treatment benefit/risk in atrial fibrillation, and minimizes the hemorrhagic risk.

## Introduction

Atrial fibrillation (AF) is the commonest cardiac rhythm disorder. The prevalence increases with population ageing [1]. The main complication is the occurrence of thromboembolic accidents, mostly cerebral ones, and they should be prevented by anticoagulant treatment. The anticoagulant treatment is based on guidelines with simplified decision making algorithms [1]. But their use should consider the hemorrhagic risk of the patient, in order to assess the benefit/risk ratio of the treatment. Studies have demonstrated the low use of Vitamin K Antagonists (VKA) in developing countries; 34.2% in Cameroun [2]; 38% in urban area, and 19% in rural area in Zimbabwe [3]. In developed countries, the rate of VKA use is 88% in the GENEVA trial [4], and 66% in the Euro Heart Survey trial [5]. Studies demonstrate that fear of hemorrhagic risk, difficulties in controlling INR, and nutritional diet imposed by the treatment, are the alleged reasons for non-prescription of VKA [6–8]. In Burkina, no study has been performed yet on the use of VKA. The aim of our study was to assess the use of VKA in the prevention of Thromboembolic risk, in AF, based on international guidelines.

## Methods

It was a descriptive retrospective study of patient's record, performed in the cardiology department from January 1st to December 31st 2011. The study involved all patients with non valvular AF. Those with documented AF on ECG and/or ECG holter were included. Echocardiography Doppler allowed the selection of patients with non-valvular AF. Thromboembolic risk was assessed through the CHA_2_DS_2_VASc score. The risk was low for a score of 0, intermediate for a score of 1, and high for a score > 2 [9]. The HAS-BLED score was used to assess the hemorrhagic risk. The risk was low for a score < 1, intermediate for a score of 2 or 3, and high for a score > 4 [10]. Assessment of anticoagulants use was about VKA in primary prevention. Indications for primary prevention were based on the guidelines of the European Society of Cardiology (ESC) [11]. Data were analyzed with the EPI-INFO7 software. Khi 2 and ANOVA were used for statistic tests. They were significant when p < 0.05.

## Results

**Frequency**: During the study period, 970 patients were hospitalized. We recorded 103 cases of AF (10.6% of hospitalized patients). AF was non valvular in 68 cases (66% of AF, and 7% of all hospitalizations). AF was permanent, chronic in 40 cases (58.8% of the cases). Table 1 shows the classification of AF.

**Table 1:** Classification of atriale fibrillation

	Frequency	Percentage
Paroxysmal	08	11.8
Persistent	08	11.8
Long Standing persistent	12	58.8
Permanent	40	17.6
Total	68	100

**Sex**: The sex ratio was 1.2, with 37 males (55.4%)

**Age**: The mean age of the population was 65.5 years old, with extremes of 26 and 99. The mean age of patients receiving VKA, was 62.9 with extremes of 26 and 87. Those without VKA treatment were 65.4 with extremes of 35 and 99 (p = 0.488). The age range 65 -74 accounted for 33.8% of the cases (n = 23). Table 2 shows the distribution of patients according to age ranges.

**Table 2:** Distribution of patients according to age ranges

	Frequency	Percentage
≤ 34	01	01.5
35 -44	05	07.4
45 -54	09	13.2
55 -64	13	19.1
65 -74	23	33.8
≥ 75	17	25.0
Total	68	100

**Residence area**: Patients were staying in Ouaga in 47 cases (69.1%); they were coming from the districts and surroundings of Ouagadougou in 21 cases (30.9%).

**Past medical history**: History of heart failure was noticed in 41 cases (60.3%). Table 3 shows the distribution of the main past medical history.

**Table 3:** Distribution of patients according to main past medical history (n = 68)

	Frequency	Percentage
Heart failure	41	60.3
Ischemic stroke	05	07.4
Coronary artery disease	02	02.9
Hyperthyroidism	07	10.3

**Assessment of thromboembolic risk**: Mean CHA_2_DS_2_VASc score was 3.8. Mean CHA2DS2VASc score in patients on VKA treatment was 4.0. Those without VKA treatment had a score of 3.8. Thromboembolic risk was high in 97.1% of the cases. Table 4 shows the assesment of thromboembolic risk. Heart failure due to systolic dysfunction was noticed in 59 cases (86.8%). Table 4 shows the distribution of the different elements of the CHA2DS2VASc score.

**Table 4:** Assesment of thromboembolic risk (n=68)

	Frequency	Percentage
**Thromboembolic risk**		
Low	-	-
Intermediate	02	02.9
High	66	97.1
**CHA**_**2**_ **DS**_**2**_ **VASC risk factor**		
Heart failure/ Left ventricular dysfunction	59	86.8
Hypertension	45	66.2
Age > 75	17	25.0
Diabete mellitus	14	20.6
Transient ischemic attack/Ischemic stroke/ Thromboembolism	23	33.8
Vascular disease	14	20.6
Age between 65-74	23	33.8
Female sex	31	45.6

**Thromboembolic complications**: We had 23 cases (33.8%) of ischemic stroke, including 5 cases of recurrence, and 14 cases of intra cavitary thrombus. The mean age of patients having ischemic stroke was 67 years old. Seventeen patients (73.9%) were older than 65 years, and six patients (26%) were older than 75 years.

**Use of VKA**: The rate of use of VKA was 35.3% (24 cases). Aspirin was used in 52 cases (76.5%). Twelve patients (17.6%) did not get antiplatelet therapy, nor VKA. Table 5 shows the factors associated to VKA prescription.

**Table 5:** Factors associated with used of VKA

	VKA use (n=24)	Risk Ratio (confident interval at 0.95)	p
Male sex (n=37)	16	1.7 (0.8 -3.4)	0.07
Residence at Ouagadougou (n=49)	19	1.5 (0.6 -3.4)	0.17
Age > 65 (n=40)	13	0.8 (0.4 -1.6)	0.29
Heart failure (n=59)	19	0.6 (0.3 -1.2)	0.16
Ischemic stroke (n=23)	14	2.7 (1.4 -5.2)	0.002
Intra cavitary thrombus (n=18)	14	3.9 (2.1 -7.1)	<0.001

**Assessment of haemorrhagic risk**: The mean HAS-BLED score was 3.5, with extremes of 1 and 7. The haemorrhagic risk was intermediate in 50% of the cases on VKA. Table 6 shows the assessment of haemorrhagic risk. Drugs interacting (Aspirin) with VKA treatment were found in 21 cases (87.5%), as part of the HAS-BLED score elements.

**Table 6:** Assesment of hemorrhaegic risk (n=24)

	Frequency	Percentage
**Hemorrhaegic risk (n=24)**		
Low	-	-
Intermediate	12	50
High	12	50
**HAS-BLED risk factor (n=24)**		
Hypertension	17	70.8
Elderly (Age > 65)	13	54.2
Ischemic stroke	12	50.0
Bleeding history or predisposition	03	12.5
Labile INR	03	12.5
Abnormal liver function	04	16.7
Abnormal renal function	10	41.7
Alcohol abuse	05	20.8
Drugs concomitantly	05	20.8

**Follow up**: We noticed 15 cases (22.2%) of deaths, one of them being secondary to a CVA complicating the VKA treatment.

## Discussion

### Classification of AF

AF was chronic permanent in 58.8% of the cases, and paroxystic in 11.8% of the cases. Thromboembolic risk, especially cerebral one, is major at the onset of AF and could reach 6.8% during the first months [9]. However, the annual risk of cerebral embolism during paroxystic or permanent AF, in the absence of anti-thrombotic treatment, is identical and estimated to 3 -5%; it is highly dependent on the CHA_2_DS_2_VASc score [12]. Naccarelli noticed that old AF were associated to a high risk of hospitalization, with an odd ratio of 3 [13].

### Age

The mean age of patients in our study was 65.5 years old. The frequency of AF increased along with age, and the age range of 65 -74 years represented 33.8% of the patients. Coulibaly and al. found a mean age of 58.9 years old in Côte d'Ivoire [14] and Ntep-Gweth in Cameroun [2] found a mean age of 65.8. Those two studies were about AF in general. In Burkina Faso, due to increase in life expectancy, and improvement of medical management of valvulopathies, there is an increase of diseases in the elderly. The rate of AF increases along with age. AF could be rare before the age of 40 years (< 0.5%), but the rate reaches 5% after the age of 65, and is more than 20% after 80 [15].

### Assessment of thromboembolic risk

In our study, 97% of the patients had high thromboembolic risk. heart failure and ischemic stroke in our study, were noticed respectively in 87.6% and 33.8% of the cases. The annual rate of ischemic stroke in patients having documented AF is 5%, which is two to seven times higher in patients without AF [16]. AF leads to super mortality due to cardiac decompensation and ischemic accidents [13]. Moreover, the presence of heart failure or ischemic stroke increases the risk of hospitalization by 6.6 and 3.1 times [17]. The mean age of patients who had ischemic stroke in our study was 67.years old. 73.8% were older than 65 years, and 26% were older than 75 years. Thromboembolic risk due to AF could increase with age, from 1.5% in the age group of 50 -59 years, to 23.5% per year in the age group of 80 -89 years [17].

### Use of VKA

The rate of use of VKA in our study was 35.3%. The rate of use in the GENEVA trial was 88% [4]. It was 34.2% in Cameroun [2], 66% in the Euro Heart Survey [5], and 38% in urban area, versus 19% in rural area in Zimbabwe [3]. The low rate of use of VKA in our study could be due to fear of hemorrhagic risk as well as difficulties in controlling INR. INR can be performed only in the two big cities of the country, named Ouagadougou and Bobo Dioulasso. The benefit of anticoagulant treatment is well demonstrated in AF. The older patients were the higher, the benefit. Meta-analysis globally show a 64% significative reduction of CVA, compared to placebo, meanwhile the preventive effect is only 22% on aspirin [16]. Therefore, the fear of hemorrhagic risk should not hinder that benefit. The hemorrhagic risk is only 1.5% per year in patients on anticoagulant. The use of CHA_2_DS_2_VASc and HAS-BLED scores helps in assessing the benefit/risk ratio of VKA in ischemic stroke. When the CHA_2_DS_2_VASc score is higher than the HAS-BLED score, the benefit of VKA treatment is higher than the hemorrhagic risk [10].

### HAS-BLED score

The mean HAS-BLED score was 3.5. In the PISTERS trial, when the HAS-BLED score was 3 and 4, the hemorrhagic risk was respectively 3.74% patient-year, and 8.70% patient-year [10]. The risk of major bleeding during the first year of oral anticoagulant treatment is 1.5%. The long course use of VKA with an INR of 2 -3, exposes to the risk of severe bleeding with an incidence of 1.5% patients-year, and fatal bleeding with an incidence of 0.3 -0.5% patients-year [10]. In our study, 97% of the patients had a HAS-BLED score > 2, and only 34% got VKA treatment. The presence of ischemic CVA, and intra cavity thrombus were statistically associated to the use of VKA. VKA could be used in our context, more for secondary prevention, than primary prevention. In the USA, only 50% of patients in need of anticoagulants are on treatment [18, 19]. The alleged reasons were hemorrhagic risk, age of patients, and monitoring of treatment. But comparative analysis in our study did not show evidence of such relation. In fact, similar studies demonstrated that the reasons for doctors not prescribing VKA, were recurrent visits, INR control, and hemorrhagic risk. Patients themselves refused VKA because of INR tests and nutritional diet related to VKA treatment [6–8]. Furthermore, in a study performed in AF patients at high thromboembolic risk, and practitioners treating the AF, patients could accept better the hemorrhagic risk compare to the thromboembolic risk, than the practitioners [6]. However, despite their obvious benefit, VKA have inter and intra individual pharmacodynamics that is difficult to predict. Therefore they need continuous monitoring, leading to a reduction of their prescription in current practice [20].

### Follow-up

We noticed 22% of deaths, one of them being secondary to haemorrhagic stroke complicating the VKA treatment. In the GENEVA trial, the rate of major bleeding was 1.5%, despite the high rate of anticoagulation (88%) [2]. To prevent such complication a better monitoring of anticoagulation should be planned in our country.

### Limits

It was a monocentric study performed on patients hospitalized in a cardiology department. This will lead to a bias in the assessment of thromboembolic risk, because ambulatory patients are not taken into account. Moreover the retrospective pattern of our study did not allow the assessment of the incidence of thromboembolic and hemorrhagic accidents.

## Conclusion

Thromboembolic complications are a major problem in the management of AF. Guidelines are well designed, but poorly used due to fear of hemorrhagic risk, and difficulties in monitoring anticoagulant treatment. Implementation of scores helps in assessing the benefit/risk ratio of oral anticoagulants, in order to reduce the hemorrhagic risk. The use of new oral anticoagulants is an alternative to minimize the risk, but there are not accessible to our populations. Involvement of general practitioners and improvement of accessibility to VKA treatment monitoring shall increase the use of anticoagulants, and improve the treatment monitoring.

### What is known about this topic

- Most complications of atrial fibrillation is the occurrence of thromboembolic accidents;
- Existence of guidelines for anticoagulants treatment;
- Low used of vitamins K antagonists in developing country.

### What this study adds

- Low used of vitamins K antagonists in primary prevention;
- Low used of vitamins K antagonists in our study;
- Poor used of guidelines due to fear of hemorrhagic risk and difficulties in monitoring anticoagulants treatment.
